# Supplementary material for: A Likelihood Approach for Real-Time Calibration of Stochastic Compartmental Epidemic Models
Source: PLoS Comput Biol. 2017 Jan 17;13(1):e1005257. doi: 10.1371/journal.pcbi.1005257 (PMC5240920; doi:10.1371/journal.pcbi.1005257)
Supplement: S1 File — (TAR.GZ) [file pcbi.1005257.s014.tar.gz › HSPH_Online-SI-Revision/MSS10/n1-extreme/n1-extreme_NEW2_table0.pdf]

1	newly 3 weeks spec	79.	72.43	{54., 86.}	12.9241	0.062934	newly 3 weeks cumul	342.	304.9	{264., 339.}	11.2164	0.0538819
2	newly 3 weeks spec	61.	49.39	{41., 58.}	19.3934	0.0978974	newly 3 weeks cumul	211.	190.45	{174., 205.}	9.93839	0.0462304
3	newly 3 weeks spec	93.	78.39	{66., 92.}	16.4839	0.0872484	newly 3 weeks cumul	270.	272.39	{241., 297.}	6.47037	0.0284458
4	newly 3 weeks spec	61.	70.3	{57., 82.}	18.3279	0.071976	newly 3 weeks cumul	180.	227.64	{204., 251.}	26.5111	0.100759
5	newly 3 weeks spec	56.	24.81	{17., 33.}	55.6964	0.369087	newly 3 weeks cumul	216.	114.02	{92., 135.}	47.213	0.282248
6	newly 3 weeks spec	54.	10.93	{0., 28.}	79.7963	1.3341	newly 3 weeks cumul	153.	113.43	{13., 206.}	41.732	0.331271
7	newly 3 weeks spec	68.	51.22	{25., 71.}	28.1471	0.182124	newly 3 weeks cumul	227.	191.77	{128., 233.}	17.1674	0.098868
8	newly 3 weeks spec	44.	40.57	{31., 49.}	14.75	0.0706727	newly 3 weeks cumul	184.	152.69	{135., 170.}	17.0163	0.082582
9	newly 3 weeks spec	65.	45.79	{20., 83.}	43.1846	0.274793	newly 3 weeks cumul	257.	204.68	{100., 290.}	26.3424	0.156539
10	newly 3 weeks spec	63.	58.69	{49., 70.}	12.3016	0.0592566	newly 3 weeks cumul	193.	208.4	{192., 225.}	11.171	0.0477219
11	newly 3 weeks spec	56.	65.72	{50., 79.}	28.3929	0.124345	newly 3 weeks cumul	197.	237.67	{216., 270.}	27.5584	0.114994
12	newly 3 weeks spec	60.	92.75	{81., 104.}	54.5833	0.186883	newly 3 weeks cumul	251.	315.96	{291., 337.}	25.9044	0.0993226
13	newly 3 weeks spec	43.	49.62	{24., 68.}	35.2558	0.152589	newly 3 weeks cumul	128.	167.02	{95., 210.}	42.6719	0.163923
14	newly 3 weeks spec	35.	51.14	{42., 61.}	46.2286	0.161072	newly 3 weeks cumul	175.	200.18	{184., 216.}	14.8229	0.059318
15	newly 3 weeks spec	59.	52.95	{43., 61.}	12.6271	0.0612555	newly 3 weeks cumul	186.	172.56	{155., 190.}	8.2043	0.0380433
16	newly 3 weeks spec	56.	58.47	{24., 77.}	25.7321	0.141723	newly 3 weeks cumul	175.	216.38	{134., 262.}	33.3257	0.136775
17	newly 3 weeks spec	48.	49.99	{37., 63.}	17.4375	0.0744761	newly 3 weeks cumul	177.	180.58	{153., 212.}	10.0226	0.0429934
18	newly 3 weeks spec	77.	75.51	{60., 87.}	10.1948	0.0462975	newly 3 weeks cumul	277.	266.01	{231., 289.}	6.68953	0.0307858
19	newly 3 weeks spec	85.	63.63	{27., 83.}	26.2	0.166746	newly 3 weeks cumul	252.	222.6	{123., 269.}	15.0794	0.0817327
20	newly 3 weeks spec	46.	31.93	{24., 39.}	30.587	0.164974	newly 3 weeks cumul	195.	120.02	{108., 132.}	38.4513	0.212011
21	newly 3 weeks spec	59.	55.59	{39., 75.}	21.9153	0.140903	newly 3 weeks cumul	155.	168.78	{136., 218.}	19.2129	0.0868565
22	newly 3 weeks spec	49.	39.71	{29., 53.}	22.5102	0.117262	newly 3 weeks cumul	172.	158.79	{131., 185.}	12.0174	0.0563088
23	newly 3 weeks spec	51.	75.06	{56., 92.}	47.2549	0.161761	newly 3 weeks cumul	194.	255.	{214., 292.}	31.9691	0.117977
24	newly 3 weeks spec	65.	71.63	{59., 85.}	14.2308	0.0570915	newly 3 weeks cumul	306.	288.42	{260., 313.}	6.84314	0.0314518
25	newly 3 weeks spec	55.	69.52	{57., 85.}	28.6182	0.106513	newly 3 weeks cumul	189.	219.01	{190., 250.}	16.8942	0.066036
26	newly 3 weeks spec	68.	52.71	{35., 69.}	23.6912	0.130488	newly 3 weeks cumul	336.	253.93	{205., 285.}	24.4256	0.125893
27	newly 3 weeks spec	39.	31.17	{19., 41.}	23.4103	0.126158	newly 3 weeks cumul	136.	113.36	{90., 140.}	18.7353	0.0935913
28	newly 3 weeks spec	51.	57.83	{42., 69.}	20.098	0.0803741	newly 3 weeks cumul	228.	215.74	{173., 245.}	9.37719	0.044997
29	newly 3 weeks spec	80.	34.61	{22., 47.}	56.7375	0.385617	newly 3 weeks cumul	289.	199.46	{151., 242.}	30.9827	0.168466
30	newly 3 weeks spec	25.	18.25	{9., 26.}	30.68	0.188088	newly 3 weeks cumul	94.	63.4	{43., 78.}	32.5532	0.184047
31	newly 3 weeks spec	69.	67.09	{53., 78.}	10.6812	0.0487775	newly 3 weeks cumul	249.	249.75	{228., 269.}	5.56225	0.0241649
32	newly 3 weeks spec	64.	63.63	{43., 79.}	18.0781	0.0928762	newly 3 weeks cumul	288.	241.87	{196., 277.}	16.1215	0.0824152
33	newly 3 weeks spec	77.	68.11	{56., 79.}	13.3117	0.0642537	newly 3 weeks cumul	224.	222.51	{204., 242.}	5.12946	0.0224283
34	newly 3 weeks spec	60.	57.91	{43., 72.}	14.8833	0.0699228	newly 3 weeks cumul	219.	217.89	{168., 246.}	9.75799	0.0444731
35	newly 3 weeks spec	68.	73.23	{61., 86.}	16.2794	0.0801888	newly 3 weeks cumul	222.	257.87	{210., 285.}	20.0315	0.0808401
36	newly 3 weeks spec	69.	60.18	{47., 75.}	16.7536	0.0850913	newly 3 weeks cumul	224.	210.13	{169., 246.}	11.2813	0.0538764
37	newly 3 weeks spec	58.	35.54	{26., 45.}	38.7241	0.232521	newly 3 weeks cumul	192.	128.05	{111., 148.}	33.3073	0.185935
38	newly 3 weeks spec	60.	51.55	{41., 61.}	15.3833	0.0759619	newly 3 weeks cumul	244.	196.34	{181., 211.}	19.5328	0.0952106
39	newly 3 weeks spec	93.	86.93	{76., 98.}	9.25806	0.0426775	newly 3 weeks cumul	314.	294.47	{269., 320.}	7.21338	0.0329769
40	newly 3 weeks spec	44.	7.25	{0., 19.}	84.3409	1.36872	newly 3 weeks cumul	165.	80.77	{17., 136.}	52.5758	0.428466
41	newly 3 weeks spec	54.	26.8	{10., 44.}	52.7778	0.388965	newly 3 weeks cumul	164.	110.34	{53., 170.}	37.1341	0.228677
42	newly 3 weeks spec	75.	71.84	{61., 82.}	9.76	0.0452042	newly 3 weeks cumul	284.	263.42	{245., 282.}	7.42958	0.0340537
43	newly 3 weeks spec	82.	75.79	{42., 92.}	15.4756	0.100843	newly 3 weeks cumul	252.	274.63	{193., 312.}	19.5198	0.0909103
44	newly 3 weeks spec	70.	62.79	{51., 73.}	12.5857	0.0606808	newly 3 weeks cumul	222.	228.41	{204., 252.}	6.92342	0.0294829
45	newly 3 weeks spec	80.	79.77	{65., 95.}	11.8875	0.0540563	newly 3 weeks cumul	274.	288.43	{254., 318.}	9.42701	0.040038
46	newly 3 weeks spec	54.	71.28	{60., 85.}	35.4444	0.143572	newly 3 weeks cumul	225.	255.96	{236., 282.}	17.4133	0.0740615
47	newly 3 weeks spec	69.	60.88	{39., 83.}	20.9855	0.1097	newly 3 weeks cumul	202.	210.53	{160., 262.}	17.3812	0.0768216
48	newly 3 weeks spec	53.	71.94	{61., 83.}	37.2453	0.135034	newly 3 weeks cumul	180.	228.56	{207., 250.}	28.1333	0.107169
49	newly 3 weeks spec	56.	59.3	{49., 72.}	13.3929	0.0561963	newly 3 weeks cumul	258.	221.69	{198., 253.}	14.686	0.0709122
50	newly 3 weeks spec	49.	19.48	{3., 35.}	60.4082	0.578433	newly 3 weeks cumul	150.	93.79	{44., 148.}	38.94	0.26402

1	newly 5 weeks spec	40.	32.74	{20., 43.}	22.9	0.124586	newly 5 weeks cumul	434.	387.09	{340., 430.}	11.0622	0.0533795
2	newly 5 weeks spec	24.	30.13	{21., 37.}	30.2083	0.112368	newly 5 weeks cumul	275.	259.16	{240., 275.}	6.16	0.0282179
3	newly 5 weeks spec	51.	48.05	{39., 60.}	14.9608	0.0940662	newly 5 weeks cumul	381.	381.95	{339., 412.}	6.26509	0.0281318
4	newly 5 weeks spec	40.	52.71	{43., 62.}	32.875	0.119893	newly 5 weeks cumul	278.	344.17	{318., 367.}	23.8525	0.0920967
5	newly 5 weeks spec	40.	11.3	{5., 17.}	71.75	0.603495	newly 5 weeks cumul	294.	141.74	{115., 167.}	51.7891	0.321679
6	newly 5 weeks spec	31.	1.38	{0., 5.}	95.5484	2.09996	newly 5 weeks cumul	228.	119.2	{13., 213.}	48.7982	0.431206
7	newly 5 weeks spec	52.	30.18	{4., 47.}	42.6923	0.403137	newly 5 weeks cumul	341.	262.07	{146., 333.}	23.3226	0.14141
8	newly 5 weeks spec	25.	25.68	{18., 32.}	18.16	0.0800491	newly 5 weeks cumul	249.	210.44	{189., 233.}	15.494	0.0745057
9	newly 5 weeks spec	29.	19.84	{1., 52.}	67.7931	0.589161	newly 5 weeks cumul	344.	255.39	{142., 408.}	34.2645	0.207071
10	newly 5 weeks spec	45.	40.07	{30., 50.}	16.3333	0.083565	newly 5 weeks cumul	286.	297.13	{282., 317.}	7.63986	0.0343726
11	newly 5 weeks spec	31.	41.87	{32., 55.}	47.6452	0.202715	newly 5 weeks cumul	268.	331.64	{301., 369.}	31.9328	0.132281
12	newly 5 weeks spec	36.	55.18	{43., 68.}	54.0556	0.182645	newly 5 weeks cumul	329.	445.15	{417., 467.}	35.304	0.130719
13	newly 5 weeks spec	32.	36.74	{14., 55.}	41.375	0.20808	newly 5 weeks cumul	184.	247.33	{130., 313.}	47.6685	0.180448
14	newly 5 weeks spec	20.	29.74	{22., 38.}	50.4	0.170566	newly 5 weeks cumul	217.	269.89	{251., 286.}	24.5023	0.094485
15	newly 5 weeks spec	32.	40.64	{32., 48.}	28.375	0.104657	newly 5 weeks cumul	268.	259.91	{237., 280.}	5.13806	0.0230684
16	newly 5 weeks spec	29.	34.28	{8., 50.}	44.	0.245033	newly 5 weeks cumul	242.	295.22	{168., 353.}	34.4876	0.145417
17	newly 5 weeks spec	42.	31.17	{20., 43.}	29.2619	0.162478	newly 5 weeks cumul	264.	251.03	{218., 284.}	9.72348	0.0446402
18	newly 5 weeks spec	51.	45.83	{32., 57.}	16.0588	0.0803015	newly 5 weeks cumul	383.	373.59	{327., 407.}	6.66057	0.0303857
19	newly 5 weeks spec	73.	39.86	{7., 59.}	45.3973	0.386144	newly 5 weeks cumul	405.	314.57	{176., 384.}	22.516	0.129789
20	newly 5 weeks spec	24.	19.29	{13., 25.}	22.7917	0.122216	newly 5 weeks cumul	257.	163.54	{149., 175.}	36.3658	0.197207
21	newly 5 weeks spec	46.	47.76	{32., 62.}	23.0435	0.177432	newly 5 weeks cumul	257.	268.64	{224., 333.}	16.8794	0.0840181
22	newly 5 weeks spec	32.	21.09	{12., 32.}	35.4063	0.215418	newly 5 weeks cumul	242.	208.81	{170., 243.}	15.3926	0.0755574
23	newly 5 weeks spec	37.	48.22	{34., 58.}	34.2162	0.124619	newly 5 weeks cumul	294.	363.43	{312., 411.}	24.8129	0.0947705
24	newly 5 weeks spec	35.	34.15	{26., 42.}	13.8	0.0630521	newly 5 weeks cumul	386.	374.4	{345., 402.}	4.93264	0.0221525
25	newly 5 weeks spec	45.	51.92	{37., 63.}	21.6889	0.0852699	newly 5 weeks cumul	280.	330.76	{294., 371.}	18.9643	0.0742403
26	newly 5 weeks spec	22.	20.11	{9., 28.}	27.3182	0.148692	newly 5 weeks cumul	403.	307.22	{240., 349.}	23.7667	0.122892
27	newly 5 weeks spec	42.	19.24	{10., 29.}	54.1905	0.36817	newly 5 weeks cumul	218.	157.36	{121., 189.}	27.9266	0.148784
28	newly 5 weeks spec	34.	36.47	{24., 47.}	21.2059	0.0923435	newly 5 weeks cumul	314.	297.87	{238., 334.}	9.09873	0.0440769
29	newly 5 weeks spec	36.	10.1	{2., 17.}	71.9444	0.680681	newly 5 weeks cumul	356.	227.86	{167., 276.}	35.9944	0.201449
30	newly 5 weeks spec	18.	12.3	{4., 19.}	34.4444	0.252037	newly 5 weeks cumul	134.	90.7	{46., 112.}	32.3134	0.187081
31	newly 5 weeks spec	42.	38.36	{28., 47.}	15.4286	0.0743728	newly 5 weeks cumul	339.	341.08	{315., 362.}	4.30678	0.0188288
32	newly 5 weeks spec	34.	35.5	{11., 49.}	33.7059	0.184753	newly 5 weeks cumul	366.	327.1	{244., 378.}	12.0546	0.0643673
33	newly 5 weeks spec	47.	53.79	{44., 64.}	17.0851	0.0665679	newly 5 weeks cumul	333.	337.85	{317., 361.}	3.93093	0.0168185
34	newly 5 weeks spec	38.	33.65	{21., 46.}	23.1842	0.121058	newly 5 weeks cumul	306.	296.07	{221., 334.}	9.91176	0.0467877
35	newly 5 weeks spec	42.	48.11	{38., 59.}	22.6429	0.12167	newly 5 weeks cumul	317.	366.26	{305., 398.}	19.6088	0.0811338
36	newly 5 weeks spec	46.	38.53	{27., 51.}	21.6304	0.115323	newly 5 weeks cumul	333.	296.29	{240., 341.}	12.2853	0.0614037
37	newly 5 weeks spec	31.	25.69	{15., 35.}	23.5806	0.194763	newly 5 weeks cumul	273.	183.61	{161., 212.}	32.7436	0.184788
38	newly 5 weeks spec	40.	31.64	{24., 39.}	22.8	0.118783	newly 5 weeks cumul	319.	268.51	{250., 287.}	15.8276	0.0754883
39	newly 5 weeks spec	46.	56.28	{46., 67.}	23.9565	0.0902428	newly 5 weeks cumul	440.	421.83	{397., 446.}	5.06136	0.0227519
40	newly 5 weeks spec	31.	0.96	{0., 0.}	96.9032	2.32141	newly 5 weeks cumul	230.	84.02	{17., 144.}	63.8435	0.55259
41	newly 5 weeks spec	33.	12.91	{0., 29.}	68.4545	0.747742	newly 5 weeks cumul	248.	142.53	{67., 224.}	45.4073	0.294629
42	newly 5 weeks spec	55.	44.03	{35., 54.}	21.0364	0.108408	newly 5 weeks cumul	401.	365.96	{340., 386.}	8.76808	0.0403829
43	newly 5 weeks spec	62.	43.94	{11., 59.}	29.7097	0.265036	newly 5 weeks cumul	378.	378.3	{217., 428.}	14.3175	0.0795486
44	newly 5 weeks spec	43.	38.08	{29., 49.}	17.2558	0.0858379	newly 5 weeks cumul	326.	314.72	{291., 342.}	5.74233	0.0262079
45	newly 5 weeks spec	45.	46.83	{33., 58.}	18.1111	0.0811556	newly 5 weeks cumul	386.	397.63	{342., 437.}	9.00259	0.0395406
46	newly 5 weeks spec	46.	43.62	{31., 55.}	18.	0.117504	newly 5 weeks cumul	335.	356.89	{325., 386.}	11.9731	0.0567446
47	newly 5 weeks spec	59.	39.1	{22., 54.}	34.3051	0.248738	newly 5 weeks cumul	320.	299.68	{224., 383.}	16.6188	0.0837574
48	newly 5 weeks spec	40.	52.15	{43., 63.}	33.175	0.123073	newly 5 weeks cumul	270.	343.28	{326., 367.}	28.6963	0.109841
49	newly 5 weeks spec	43.	36.14	{26., 45.}	19.3023	0.113373	newly 5 weeks cumul	341.	304.04	{277., 350.}	11.9413	0.0571821
50	newly 5 weeks spec	26.	6.22	{0., 16.}	76.1538	1.11481	newly 5 weeks cumul	211.	110.97	{46., 172.}	47.654	0.340224

1	newly 60 weeks cumul	525.	443.63	{365., 502.}	15.64	0.077265						
2	newly 56 weeks cumul	368.	346.14	{306., 378.}	7.38043	0.0343853						
3	newly 58 weeks cumul	577.	519.35	{470., 574.}	10.5563	0.0512988						
4	newly 57 weeks cumul	490.	541.93	{506., 581.}	11.4673	0.0469551						
5	newly 50 weeks cumul	415.	162.32	{134., 197.}	60.8867	0.41273						
6	newly 55 weeks cumul	355.	119.84	{13., 213.}	66.2423	0.61743						
7	newly 59 weeks cumul	479.	354.43	{147., 462.}	26.0564	0.174822						
8	newly 53 weeks cumul	332.	287.07	{251., 313.}	13.5512	0.0645781						
9	newly 60 weeks cumul	427.	301.81	{148., 563.}	44.2693	0.268973						
10	newly 53 weeks cumul	451.	434.41	{424., 462.}	4.85366	0.025648						
11	newly 59 weeks cumul	345.	461.31	{394., 517.}	43.1855	0.1696						
12	newly 60 weeks cumul	420.	599.44	{531., 634.}	42.7238	0.15323						
13	newly 53 weeks cumul	478.	388.68	{196., 489.}	20.159	0.132482						
14	newly 57 weeks cumul	250.	355.19	{338., 374.}	42.156	0.151861						
15	newly 52 weeks cumul	342.	445.29	{416., 473.}	30.2018	0.114165						
16	newly 59 weeks cumul	393.	382.2	{185., 473.}	20.631	0.114516						
17	newly 56 weeks cumul	488.	342.74	{281., 400.}	29.9713	0.158693						
18	newly 59 weeks cumul	532.	511.87	{414., 558.}	7.77256	0.0368745						
19	newly 60 weeks cumul	563.	427.89	{199., 546.}	24.0586	0.152252						
20	newly 53 weeks cumul	327.	223.06	{200., 244.}	31.7859	0.167281						
21	newly 56 weeks cumul	478.	520.06	{420., 596.}	19.2427	0.102374						
22	newly 57 weeks cumul	358.	254.68	{201., 311.}	28.8603	0.153539						
23	newly 59 weeks cumul	412.	501.61	{413., 577.}	23.5413	0.0899877						
24	newly 60 weeks cumul	448.	444.28	{412., 472.}	3.99107	0.0176141						
25	newly 57 weeks cumul	488.	503.1	{413., 560.}	9.53279	0.0412277						
26	newly 59 weeks cumul	451.	333.37	{254., 380.}	26.082	0.137196						
27	newly 54 weeks cumul	436.	220.21	{160., 288.}	49.4931	0.307152						
28	newly 58 weeks cumul	442.	407.44	{308., 458.}	9.97738	0.0509972						
29	newly 55 weeks cumul	420.	239.07	{172., 289.}	43.0786	0.25253						
30	newly 50 weeks cumul	194.	148.48	{61., 194.}	24.3299	0.152154						
31	newly 59 weeks cumul	419.	440.11	{405., 469.}	7.08592	0.0300708						
32	newly 59 weeks cumul	414.	426.67	{253., 504.}	21.8575	0.1042						
33	newly 55 weeks cumul	564.	553.61	{524., 579.}	3.2961	0.0146654						
34	newly 58 weeks cumul	433.	386.45	{288., 470.}	14.9861	0.0739125						
35	newly 59 weeks cumul	467.	522.5	{444., 568.}	17.3019	0.075627						
36	newly 56 weeks cumul	462.	407.25	{306., 474.}	13.2792	0.0670161						
37	newly 51 weeks cumul	413.	289.26	{253., 329.}	29.9613	0.17361						
38	newly 56 weeks cumul	406.	359.26	{326., 408.}	11.9507	0.0565345						
39	newly 59 weeks cumul	558.	595.45	{579., 613.}	7.02688	0.0294808						
40	newly 57 weeks cumul	342.	85.12	{17., 144.}	75.152	0.721232						
41	newly 52 weeks cumul	369.	170.98	{71., 246.}	56.9322	0.404089						
42	newly 60 weeks cumul	505.	493.18	{431., 536.}	6.62574	0.0302403						
43	newly 59 weeks cumul	545.	496.6	{225., 569.}	11.3908	0.0791666						
44	newly 57 weeks cumul	430.	421.7	{393., 448.}	4.89767	0.0228855						
45	newly 60 weeks cumul	569.	519.08	{412., 585.}	10.3902	0.0516167						
46	newly 57 weeks cumul	485.	493.35	{423., 540.}	10.1959	0.0541896						
47	newly 58 weeks cumul	544.	406.73	{278., 524.}	25.2702	0.145491						
48	newly 58 weeks cumul	460.	548.74	{526., 582.}	22.7478	0.0916835						
49	newly 60 weeks cumul	475.	409.71	{375., 455.}	13.7453	0.0680332						
50	newly 58 weeks cumul	361.	120.77	{46., 193.}	66.5457	0.544445						
